# Household Pharmaceutical Waste Management Practices in the Johannesburg Area, South Africa

**DOI:** 10.3390/ijerph19127484

**Published:** 2022-06-18

**Authors:** Benele K. Magagula, Isaac T. Rampedi, Kowiyou Yessoufou

**Affiliations:** Department of Geography, Environmental Management & Energy Studies, University of Johannesburg, P.O. Box 524. Auckland Park, Johannesburg 2092, South Africa; magagulabk1@gmail.com (B.K.M.); kowiyouy@uj.ac.za (K.Y.)

**Keywords:** pharmaceutical wastes, drugs, pollution, survey, awareness, storage, disposal

## Abstract

Pharmaceutical wastes are expected to increase given the increasing population growth rates and rapidly rising economic burden of human diseases. This challenge calls for appropriate measures for the management of such hazardous wastes. The purpose of this survey was to document and investigate existing practices for the handling, storage, and disposal of household pharmaceutical wastes (HPWs) in the Johannesburg area. Primary data were collected via online surveys with self-administered questionnaires completed by respondents. The research found that 77% (*n* = 286) of respondents claimed some knowledge about HPWs. Types of medicines that contributed to HPWs included painkillers or analgesics (73%, *n* = 270) and drugs for treating colds and flu-related illnesses (52%; *n* = 193). Although there were a few exceptions, the respondents generally exhibited strong disagreements with environmentally unfriendly and health-threatening disposal practices. Moreover, most participants were willing to return expired medicines to pharmacies (40.7%, *n* = 151), whereas only 8.6% (*n* = 32) opposed this solution. Awareness levels tended to vary with employment status, educational qualifications, and place of residence. However, place of residence and household size did not correlate with types of pharmaceutical waste. Additionally, the study found that education attainments significantly influenced the willingness of respondents to return pharmaceutical wastes. Finally, there were no significant differences amongst respondents in terms of waste disposal practices. Altogether, the findings suggest the need for targeted efforts to bring about sustainable waste management at a household level.

## 1. Introduction

The world human population exceeded 7 billion in the year 2017, and it is likely to reach 9.7 billion by 2050; therefore, more pharmaceutical products and medicines will be needed to deal with the growing economic and public health burdens of human diseases [1]. For example, since December 2019, there has been an increased global demand for the manufacturing of ventilators and drugs needed to treat patients affected by the COVID-19 pandemic. Nearly 1.42 trillion USD was spent on medicines in 2021, an increase from 887 billion USD in 2010 [2]. The figure is expected to reach about 1.8 trillion USD during the year 2026 [2]. Similarly, in India, it is projected that the sales of over-the-counter medicines are expected to increase from approximately USD 125 billion in 2016 to approximately USD 273 billion by the year 2024 [3]. Inevitably, with such high consumption rates of medicines, there will be an increase in pharmaceutical wastes from pharmaceutical companies and pharmacies where drugs and associated products are manufactured and dispensed, including hospitals and health-care centres, and even in households where most people keep medicines to treat their ill-health and wellness problems.

Pharmaceutical wastes entail expired drugs and nutraceuticals, unused medicines, discarded wellness products, and even medicines that are no longer needed because patients have either died or have recovered from diseases [3,4]. This waste category also includes all related accessories such as bandages, plastic spoons and related utensils, syringes, injection needles, elastoplasts, and even solid materials such as packaging plastic and paper materials [5]. A household waste stream of this type is potentially hazardous and can be health-threatening if it is not handled, stored, and disposed of properly. It can also escape into the different areas of the environment, such as water bodies, vegetation, air and land surfaces [3,6].

There is growing evidence that some of the pharmaceutical wastes, including well-known drugs, are now circulating in the environment, thus threatening to disrupt the functioning of natural ecosystems and human health. While the presence of such drugs in the natural environment may be partly ascribed to the release of their metabolised and unmetabolized fractions via normal excretion from the human body, a certain proportion of them is traceable to inappropriate disposal methods. For example, metformin, a drug used to treat diabetic patients, has been reported in municipal wastewaters due to both normal excretions and inappropriate disposal via sinks and toilet drains [7]. Similarly, anti-retroviral drugs (so-called ARVs) for treating HIV/AIDS patients have been found in both surface water bodies and wastewater treatment plants [8,9]. Due to this growing trend, it is becoming expensive for water purification companies and water management schemes to remove these drugs, as well as their metabolites, from polluted water bodies [10,11,12]. Additionally, children have fallen victim to easy-to-reach medicines because they are improperly kept at home, causing needless substance poisoning, physiological damage, and even death in some instances [13,14]. At other times, pharmaceutical wastes are simply mixed with household general waste destined for municipal landfill sites, thus posing another undesirable pathway that releases harmful chemicals into the natural environment [6,15,16].

Such environmentally unsustainable and unsafe practices show that medicines and all of the associated pharmaceutical wastes emanating from them require proper handling, storage as well as safe disposal. Unfortunately, these precautionary measures are lacking in some households, particularly in the developing countries due to poor accessibility to sound medical advice and limited knowledge. Another factor is that, in most African countries, the processes required to handle pharmaceutical wastes at household levels are not legislated. Moreover, pharmaceutical wastes inside households may accumulate for a variety of reasons, such as (1) improvements in the patient’s medical conditions, (2) over-supply of medicines by pharmacies, and the use of excessive packaging materials [17,18]. In a study conducted by Amod et al. [16] on 200 randomly sampled adults, nearly 63% of their respondents disposed of their expired medicines into municipal waste bins, 17% washed them down kitchen sinks, 5% flushed them into toilet drains, while about 4% returned them to pharmacies.

Regardless of the importance of these findings and the threat to human wellness and environmental quality, South Africa is lagging behind in empirical studies on the management of pharmaceutical wastes in the hospitals, community clinics, and health care centres, as well as households and communities in general. Overall, households constitute one of the major disposal pathways through which pharmaceutical toxins can reach various environmental media [5]. Such a gap in the literature called for the present survey, which aims to document and investigate practices in the handling, storage and disposal of pharmaceutical wastes amongst households in the Johannesburg area in South Africa. Specifically, the survey documented (i) the composition and people’s awareness of household pharmaceutical wastes (HPWs), (ii) their storage and disposal practices, (iii) household willingness to return unwanted or unused medicines back to local pharmacies, and (iv) four hypotheses to identify the determinants of people’s behaviour in relation to the previous three (i–iii) research objectives. This investigation is important, especially as the City of Johannesburg has embarked on a compulsory household waste sorting and recycling program since 1 July 2018. This new waste minimization approach was necessitated by the adoption of the new City of Johannesburg Waste Management By-Laws [19]. The recycling programme covers about 570,312 households and nine depots in the City of Johannesburg [20].

## 2. Study Area, Materials and Methods

### 2.1. Description of Study Area

**Figure 1:**
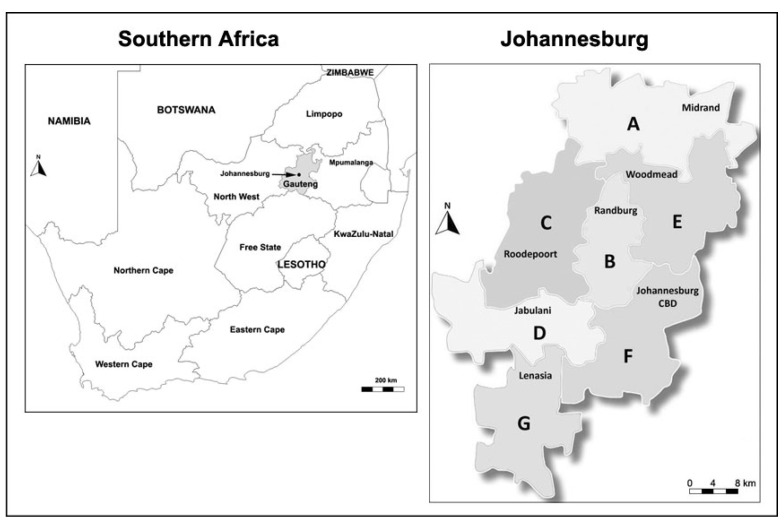
Geographical location of Johannesburg and the different regions of this city. (A = Region A; B = Region B; C = Region C; D = Region D; E = Region E; F = Region F; and G = Region G).

The study area comprised households in and around the City of Johannesburg Municipality in the Gauteng province (Figure 1). Johannesburg is the largest city in South Africa and is part of the Gauteng City Region. According to Statistics South Africa [21], the mid-year 2019 population estimate for the Gauteng province was 15,176,115 inhabitants. Based on the World Population Review [22], Johannesburg’s population is estimated at 5,782,747 inhabitants spread across 130 municipal wards. The city is also delineated into seven different regions named Region A–Region G. The 2011 Census reported about 1,434,856 households in this municipality, with an average of 2.8 persons per household. 

### 2.2. Study Design and Research Methods

In order to address the aim of the present study, a quantitative survey research design was adopted. Quantitative surveys have been extensively by various researchers [23,24,25,26] in waste management research. A survey is a systematic method of gathering primary data from samples of entities that belong to a larger population [27]. Thus, the ‘ultimate goal is to learn more about a large population by surveying only a sample of that population’ [28]. The targeted unit of analysis in this survey was respondents representing residential households in Johannesburg (South Africa), the main purpose being to understand management practices associated with their HPWs.

### 2.3. Questionnaire Design

An online digital questionnaire was designed to collect primary data. The instrument had four main sections, and they are summarized as follows:

- Section A (demographical aspects);
- Section B (awareness of HPWs);
- Section C (storage and disposal practices);
- Section D (willingness to return unused/expired or discarded medicines and associated accessories back to local hospitals and pharmacies).

Whereas some of the questions in the questionnaire were straightforward or close-ended, thus requiring only a yes or no answer, others were based on a Likert scale. With a Likert scale, it is possible to rate the extent to which respondents are agreeing or disagreeing with formulated statements [5,29,30,31]. For example, based on a Likert scale of 1 to 5, where 1 = Strongly Disagree, 2 = Disagree, 3 = Neither Disagree nor Agree, 4 = Agree and 5 = Strongly Agree, respondents in the present survey were requested to indicate the extent to which they agreed or disagreed with each of the statements on household storage and disposal practices of HPWs and their willingness to return such wastes to hospitals and pharmacies. Furthermore, the internal validity of the formulated questions was pre-tested by means of the Cronbach alpha (α) coefficient, whose formula is depicted below:

(1) $$\alpha =(N\cdot c)/[\overset{-}{v}+(N-1)\cdot \overset{-}{c}]$$

where N denotes the number of scale or items, c-bar represents the average inter-item covariance among the scale items, and v-bar is the average variance [32].

The value of the coefficient ranges from 0 to 1, and coefficient values closer to 1 show a higher internal consistency of the variables in the scale. In the present study, this coefficient was 0.68, thus indicating that the questionnaire was reliable.

### 2.4. Primary Data Collection

Primary data were collected online by means of a self-administered questionnaire completed by respondents during the August–November 2021 period. In the questionnaire, respondents were asked questions that allowed them to share (i) the types of HPWs they generate at home and their level of awareness on this waste stream, (ii) their practices regarding the storage and disposal of HPWs, and (iii) their willingness to return their unused medicines back to local pharmacies.

Potential respondents above the age of 18 years old were students at the University of Johannesburg (Auckland Park Kingsway Campus). They were contacted via digital communication applications or platforms, such as e-mail, WhatsApp, and Telegram. In total, 434 individuals were contacted, of which only 371 responded, thus giving a response rate of about 85%.

The students who were willing to consent and participate received an online digital consent form along with a digital questionnaire (i.e., Google Form). The students were also asked to send the initial digital link and questionnaire to other people they knew living in the Johannesburg area, thus broadening the initial base of respondents by means of snowball leads or chain referrals. While many contacts were made during the primary data collection, it is imperative to acknowledge that the approach we followed brought some inevitable limitations to the research. Thus, the respondents without access to electronic devices were inevitably excluded from the sampling framework. Consequently, the results generated may not be widely generalizable to all households in Johannesburg. Despite this limitation, the findings revealed important patterns regarding HPW practices among the youthful, educated and the digitally connected population in the City of Johannesburg.

### 2.5. Data Analyses

Especially for the first three research objectives, to determine (i) the types of HPWs they generate and their level of awareness about this waste stream, (ii) people’s practices for the storage and disposal of HPWs, and (iii) to document people’s willingness to return back unused medicine to local pharmacies, descriptive statistics and the percentages (%) of respondents were calculated. However, to test the following four hypotheses, an analysis of variance (ANOVA) was conducted on the collected data:

-
  **Hypothesis** **1**. *Household awareness about pharmaceutical waste is related to the demographical properties of households*.
-
  **Hypothesis** **2**. *There are no statistically significant differences in the various classes of pharmaceutical wastes according to the income level, place of residence, and household size of the respondents.*
-
  **Hypothesis** **3**. *Household willingness to return pharmaceutical wastes does not vary according to the age, education, gender and place of residence of respondents*.
-
  **Hypothesis** **4**. *There are statistically significant differences amongst respondents in terms of their selection of specific waste disposal waste practices.*

Factors that were significantly associated with household waste disposal practices were also selected. The *p* values were set at 0.05.

### 2.6. Ethical Procedures

As far as ethical protocols are concerned, the Code for Academic and Research Ethics of the University of Johannesburg (UJ) was duly complied with, and the research project was approved with the following reference: 2021-04-01/Magagula/Rampedi. The university’s code of conduct makes the following provisions mandatory to ensure that any risk of dealing with internal or external stakeholders in the primary data collection phase and beyond is significantly reduced, if not eliminated:

- Employees, students, and affiliates must always respect the rights of research participants to freedom, dignity, privacy (including the right to anonymity), and bodily and psychological integrity;
- Research partners and associates may be used as research participants only if they have given their written and informed consent to become participants in a research project;
- Commencement/execution of research projects is dependent on the adherence to all government and UJ regulations related to the COVID-19 pandemic, thus preventing the risk of viral infections and associated distress.

## 3. Results and Discussion

### 3.1. Demographic Attributes of Respondents

Of the total number (*n* = 371) of respondents in the survey, 72.8% (*n* = 270) were men and 27.2% (*n* = 101) were women. Other demographical characteristics are depicted in Table 1. The majority (80.6%, *n* = 299) of respondents were in the 20–29 years category. This outcome is traceable to the selection and sampling of respondents, especially as the initial contacts for the survey were mainly students at the University of Johannesburg. Besides this main group, nearly 12% of respondents were in the 30–39 years category, while other age groups were less represented. In terms of employment status, 52.6% (*n* = 195) were students enrolled at various academic institutions in South Africa, meanwhile 25.9% (*n* = 96) were employed full-time. The proportion of those who were working part-time (8.1%, *n* = 30) or unemployed (7.8%, *n* = 29) was nearly the same. Based on the highest academic qualification achieved, most of the respondents were not only literate but had acquired important academic qualifications. For instance, the percentage of those who had completed a bachelor’s university academic program was 32.1% (*n* = 119) whereas those who had postgraduate degrees amounted to 27.2% (*n* = 101).

Regarding annual income levels (Table 1), a relatively large proportion (40.7%, *n* = 173) of respondents had income levels of up to ZAR 50,000 (USD ~3160), whilst 27.1% (*n* = 115) had income levels between ZAR 100,000 (USD ~6320) and ZAR 300,000 (USD ~18,960). As further shown in Table 1, 18.4% (*n* = 78) indicated that they had incomes between ZAR 301,000 (USD ~19,023) and ZAR 500,000 (USD ~31,600). The smallest proportion of the respondents (3.3%, *n* = 14) had incomes ranging from 750,000 (USD ~47,400) to ZAR 1,000,000 (USD ~63,200). On the whole, the majority of residents in Johannesburg had an income of less than ZAR 500,000 (USD ~31 600). The statistics provided by the City of Johannesburg Metropolitan Municipality [33] indicate that about 57% of the residents in Johannesburg live on less than ZAR 400,000 (USD ~25,280) per annum [33].

In terms of household types, the majority (56.3%, *n* = 209) of respondents lived in individual or privately owned households, while 16.2% (*n* = 60) resided in flats or rented apartments (Table 1). By contrast, households located on the estates (6.7%, *n* = 25) and in communes (3.2%, *n* = 12) were less frequent. Nearly half (46.6%, *n* = 173) of the respondents lived in households occupied by 4–6 persons, and this was closely followed (38.5%, *n* = 143) by households with 1–3 persons (Table 1). Larger households with 7–9 persons accounted for 10.5% of respondents, while households occupied by more than 9 persons were very few (4.3%).

### 3.2. Awareness and Types of HPWs

**Figure 2:**
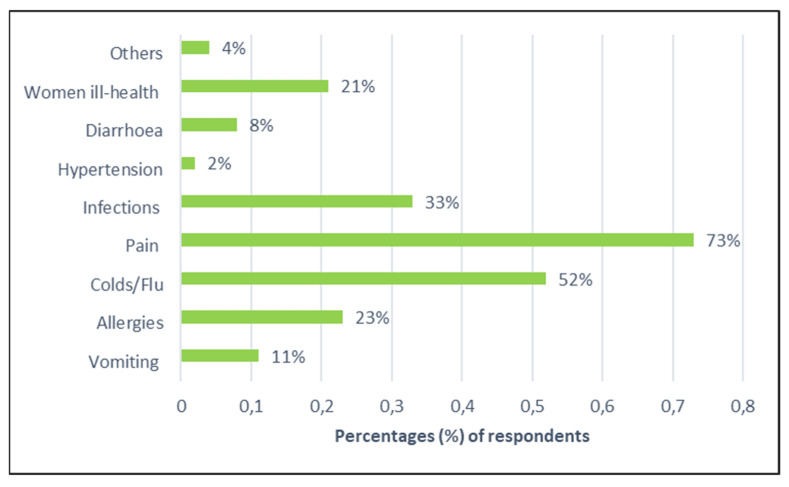
Proportions of respondents according to medicines used in their homes.

In terms of awareness about HPWs, 77% (*n* = 286) of respondents claimed to have some knowledge of such waste materials, while 23% were not aware. Therefore, investigating the different types of medicines kept at home is important as far as their contribution to the generation of HPWs is concerned. In Figure 2, the proportions of respondents who mentioned specific types of medicines used and kept in their homes are indicated. Most respondents mentioned the use of painkillers or analgesics (73%, *n* = 270) to reduce bodily pain and medicines applied to treat colds and flu-related illnesses (52%; *n* = 193). Another important class of medicines was anti-allergic drugs (23%, *n* = 85), medications for women’s health (21%, *n* = 75), and antibiotics (33%, *n* = 122) for treating various types of infections. These findings bear some similarities with some previous studies. For example, analgesics were the most frequently (46%, *n* = 115) reported medicines amongst households in Mauritius [34] as well as in the community cross-sectional survey (29%, *n* = 149) conducted in the Tigray Region of Northern Ethiopia [35]. Similarly, in Cape Town (South Africa), about 64% (*n* = 104) of respondents reported painkillers as part of unused medicines in their homes [36]. Another commonly reported group of household drugs are antibiotics, which were documented in various studies, thus denoting their prevalence in many households [35,37,38].

Amongst other types of wasted medicines, unused pills or tablets were disposed of by 29.4% (*n* = 109) of the respondents, which is relatively low compared to the results from other studies. For instance, as much as 70% (*n* = 354) of respondents mentioned unused tablets amongst some households in the Ethiopian Tigray region [35]. Such high proportions of respondents can be ascribed to the high number of over-the-counter drugs that are frequently being bought nowadays without any medical prescriptions. Furthermore, a very large proportion (96%; *n* = 359) of respondents refuted the contribution made by unused portable health monitoring devices to the household waste stream. Such items included blood pressure or blood sugar monitoring machines and thermometers, probably because they are used for chronic medical conditions. Such utilization means that they can be kept for longer periods at home without being part of the pharmaceutical waste stream up until they are eventually discarded. The same result was also recorded for unused liquid-based medicines (84.6%, *n* = 314) and unused injections (96.5%, *n* = 358). Lastly, apart from wasted drugs, Table 2 also indicates other waste items, such as the packaging materials associated with pharmaceutical products. These items were widely acknowledged by respondents in the present survey: small paper bags (42.6%), plastic bottles (49.1%), and small plastic bags (67.9%). Such packaging waste materials were not considered or mentioned in several studies conducted in South Africa [8,9] and even abroad [39,40,41].

### 3.3. HWS Storage and Disposal Practices

In Table 3, the results of the degree to which the respondents agreed or disagreed with statements on specific storage methods are summarized according to a Likert scale. To a larger degree, the majority of respondents did not store their pharmaceutical products according to the different storage options presented in the survey, although there were few exceptions. For example, 41% (*n* = 152) of respondents strongly disagreed with the statement ‘I store them inside a fridge’, with a further 10.5% (*n* = 39) of respondents disagreeing with the same statement. This high level of disagreements suggests some malpractices in the storage of medicines in the present study. According to drug manufacturers, medicines must be stored under a certain temperature profile and moisture conditions, with no exposure to light or accessibility to children. Therefore, violating these storage conditions poses a serious health risk to both patients and children within households. Similarly, improper storage practices may affect the clinical efficiency of medicines in a negative manner, apart from their potential contribution towards the generation of medicinal wastes [42,43,44]. Nonetheless, close to 191 (51%) respondents agreed that they stored their medications inside medicine boxes, which is good practice as long as they are not accessible to children and the vulnerable.

Furthermore, the respondents were asked to indicate how they disposed of their pharmaceutical wastes from households. The results are shown in Table 4. Even though there were few exceptions, the respondents generally exhibited strong disagreements with environmentally unfriendly and health-threatening disposal practices. Just over half (52.6%, *n* = 195) of the respondents did not flush their unused medicines in kitchen sinks and toilet drains, although a few (11.3%, *n* = 42) of them used this method. This kind of disposal practice has been widely criticized in many countries as one of the prime pathways for the presence and dissemination of medication compounds into the environment [5,45,46,47,48,49]. Once released into the environment, discarded drugs can negatively affect the development of biological species. In a recent study, it was established that the release of the drug diclofenac into the environment was responsible for renal failure amongst vultures in South East Asia, while trace amounts of ethinylestradiol derived from over-the-counter medicines is impairing the sexual maturity and the feminization of fish species in some of the European water bodies [49,50]. To a greater extent, amongst the respondents in the present survey, unused medicines were not given to sick people for re-use (72.5%, *n* = 269), which is good practice, although they were also not returned to hospitals and pharmacies (68.2%, *n* = 253) due to the lack of take-back programs in South Africa. Furthermore, 235 (~63%) respondents agreed to varying degrees that they disposed of their medicine waste by mixing it with their general household domestic waste. This latter finding is similar to the results from the industrial area of Malaysia, where 63.1% of their respondents simply discarded their medicinal wastes into domestic rubbish bins without any segregation [5]. Lastly, keeping unwanted medicines indefinitely at home was strongly refuted by most (42.3%, *n* = 157) respondents, thus suggesting that once medicines were used or had expired, they were disposed of unless the patients were still undergoing treatment.

In view of the negative environmental and ecological impacts caused by the release of HPWs into the environment, pharmaceutical take-back schemes are widely regarded as a potential solution to reduce these impacts, although their feasibility and success differ amongst countries [51,52,53]. In the present survey, the household willingness for participation in pharmacy take-back programs was estimated (Table 5). The results indicated that most of the respondents expressed a willingness to participate in such schemes (Table 5) although there is currently no national regulatory framework in South Africa to enforce such programs. Strong agreements amongst the respondents were expressed for the following suggestions, and they were given in the following order of magnitude: (1) returning expired medicines to pharmacies (40.7%, *n* = 151), (2) highly recommending such programs to other people (42%, *n* = 156), and (3) a willingness to follow any helpful advice given by health care providers for the safe disposal of such wastes (44.5%, *n* = 165). Only a few respondents strongly agreed with the statement that they were not doing anything to improve the situation (8.6%, *n* = 32), thus expressing their preparedness to maintain the status quo. By contrast, 42% (*n* = 156) of respondents were prepared to recommend any future take-back schemes to other people even, though such programs do not exist yet. This finding demonstrates the need to develop a national framework in South Africa that can guide the handling and disposal of HPWs at the household level.

### 3.4. Testing of Hypotheses Based on ANOVA

As mentioned earlier, several hypotheses were formulated in order to shed more light on the association between the perceptions of HPWs by respondents according to some of their demographical characteristics. Each hypothesis is analysed below according to the data generated from the ANOVA.

**Hypothesis 1**.

This study tested the hypothesis that household awareness about pharmaceutical wastes is significantly related to the demographical properties of respondents. Such properties entailed their gender, age, income level, educational level, employment status, place of residence, household size and household type. The results from the ANOVA are presented in Table 6. It was found that awareness of pharmaceutical wastes was not significantly related to demographic variables, such as gender (*p* = 0.969 > 0.05), age (*p* = 0.074 > 0.05), income level (*p* = 0.369 > 0.05), household type (*p* = 0.412 > 0.05) and household size (*p* = 0.938 > 0.05). However, awareness was found to be significantly related to employment status (*p* = 0.011 < 0.05), educational qualifications (*p* = 0.014 < 0.05), and place of residence (*p* = 0.014 < 0.05). Thus, awareness levels tended to vary by employment status, educational levels and place of residence, whilst other demographic characteristics (gender, age, income level, household size and household type) exhibited no influence on awareness levels.

**Hypothesis 2**.

Regarding this hypothesis, there were statistically significant differences in the mean scores representing the various classes of HPWs in terms of income levels (*p* = 0.039 < 0.05; Table 7), meaning that classes of pharmaceutical wastes vary according to the income levels of respondents. On the contrary, the results show that there were no statistical differences in the means for the different classes of pharmaceutical wastes and places of residence (*p* = 0.534 > 0.05) and household sizes (*p* = 0.078 > 0.05). This means that there was homogeneity in the sample in terms of places of residence and household sizes. Thus, places of residence and household sizes were not connected with the different classes of HPWs.

**Hypothesis 3**.

The ANOVA test confirmed that household willingness to return pharmaceutical wastes was not a function of gender (*p* = 0.250 > 0.05), age (*p* = 0.863 > 0.05) and places of residence (*p* = 0.567 > 0.05) (Table 8). This implies that there were no statistically significant differences between the mean scores of the variables involved. Thus, places of residence, age, and gender did not influence the willingness of households to return pharmaceutical wastes in the study area. However, the results indicated that household willingness to return pharmaceutical wastes was statistically related to level of education (*p* = 0.017 < 0.05). This finding raises the need to increase willingness via appropriate educational interventions where weaknesses or shortfalls in this practice exist.

**Hypothesis 4**.

Table 9 shows the results of the ANOVA to test whether there were statistically significant differences amongst respondents in terms of pharmaceutical waste disposal practices. The test statistics for all the constructs on waste disposal practices, except for “I throw it away into household domestic waste bin” (*p* = 0.000 < 0.05), “I flush it down the toilet drain” (*p* = 0.030 < 0.05) and “I return it to hospitals and pharmacies” (*p* = 0.000 < 0.05), exhibited *p*-values greater than 0.05, thus demonstrating no significant statistical differences amongst respondents in terms of waste disposal practices.

## 4. Conclusions, Implications and Recommendations

Given the different environmental pollution and health risks associated with the improper management of expired medicines, associated accessories, and their packaging, the prime purpose of the present study was to document and investigate different practices for the handling, storage and disposal of household pharmaceutical wastes (HPWs) in the Johannesburg area.

The study revealed that most (77%) respondents were aware of HPWs, and the most common medicines kept within households were painkillers (73%), medicines to treat colds and flu-related illnesses (52%) and antibiotics to treat other infections (33%). This household waste stream also comprised small plastic bags (67.9%), soft cardboard boxes (51.8%), and plastic bottles (49.1%), as well as typical medicine waste items, such as unused pills, injections, topical products and health-monitoring devices.

The survey revealed the different practices of storing medicines at household level. Whereas most methods were strongly refuted by the majority of respondents, close to 50% (*n* = 191) of them indicated that their medicines were stored within medicine boxes. Aside from this storage method, special efforts to store medicines in line with the instructions given by drug manufacturers were seldom made by respondents, thus explaining why some medicines ended up in household waste, which was then disposed of in municipal landfill sites. However, if the amounts of drugs that end up in household waste are to be significantly reduced within households, it is necessary to follow the precautions given when they are dispensed by pharmacies, thus maintaining their clinical efficiency and minimizing their potential wastage.

The most common method for the disposal of unused medicines was mixing them with other household wastes despite their hazardous nature. Close to 235 (63%) respondents agreed that they used this method based on the Likert scale applied in this study. Although this finding bears some resemblance with the findings of previous studies, it is imperative to state that our results were influenced to some extent by the demographical characteristics of the respondents. For instance, almost 80% (*n* = 299) of respondents were aged 20–29 years, implying that this may affect the type of drugs they use and, consequently, their management methods. The majority of respondents strongly disagreed with disposal methods, such as keeping medicines indefinitely in households (42.3%), throwing them outside (55%), burning them (61%), or returning them to local pharmacies and hospitals where they were dispensed (68%). Although the findings show that these methods are not widely practiced by respondents, the lack of household waste segregation (33.4%) should be a source of environmental concern as this allows for final landfill disposal.

According to the results showing the different degrees of willingness to return unused medicines and associated accessories to hospitals and pharmacies, most respondents expressed a positive disposition towards playing a meaningful role in such medicine take-back schemes. However, the implementation of such programs in many developing countries is fraught with implementation pitfalls due to a lack of environmental awareness, necessary infrastructure, and willingness to pay for such programs [41,54,55,56]. This constraint also applies to South Africa, where there is no well-defined national legislative and regulatory framework to deal with discarded HPWs in a sustainable manner.

Lastly, a number of hypotheses were tested using the ANOVA. Firstly, it was established that an awareness of pharmaceutical wastes is not significantly related to gender, income level, household type, and household size, although it was significantly related to employment status, educational qualifications, and places of residence. Thus, important factors to enhance the awareness of HPWs would be the introduction of targeted educational interventions at various settings, including workplaces, educational institutions, and places of residence. Secondly, there are statistically significant differences between pharmaceutical wastes specified by respondents and their income levels. This is because income plays an important role in the buying of medicines and associated products; therefore, it is not surprising that classes of HPWs were positively correlated with different income categories. Thirdly, whereas household willingness to return pharmaceutical wastes is not a function of variables such as gender, age and places of residence, the level of education was statistically related to household willingness to return unused or expired medicines. This conclusion raises the need to increase willingness to participate in the take-back programs by means of appropriate educational interventions where they do not exist. Finally, for all constructs considered to characterise waste disposal practices amongst the respondents, statistically significant differences were found only for statements such as “I throw it away into a household domestic waste bin”, “I flush it down the toilet drain” and “I return it to hospitals and pharmacies”, thus indicating similarities and dissimilarities in the disposal of such wastes.

## Figures and Tables

**Table 1:** The demographical characteristics of respondents.

Variables	Category	Households	
		F	%
Age range	20–29	299	80.6
	30–39	44	11.9
	40–49	11	3.0
	50–59	12	3.2
	60–65	4	1.1
	>65	1	0.3
	Total	371	100.0
Employment status	Full-time	96	25.9
	Part-time	30	8.1
	Unemployed	29	7.8
	Self-employed	14	3.8
	Student	195	52.6
	Other	7	1.9
	Total	371	100.0
Level of education	Some primary school	1	0.3
	Some high school	7	1.9
	Matric	98	26.4
	Post-matric diploma/certificate	45	12.1
	Bachelor’s degree	119	32.1
	Postgraduate degree	101	27.2
	Total	371	100.0
Income levels	ZAR 0-ZAR 50,000	142	38.3
	ZAR 100,000-ZAR 300,000	97	26.1
	ZAR 301,000-ZAR 500,000	73	19.7
	ZAR 500,001-ZAR 750,000	29	7.8
	ZAR 750,001-ZAR 1,000,000	14	3.8
	>ZAR 1,000,000	16	4.3
	Total	371	100.0
Household types	Private house	209	56.3
	Town house	20	5.4
	Flat/apartment	60	16.2
	Estate	25	6.7
	Commune	12	3.2
	Retirement village/old age home	7	1.9
	Other	38	10.2
	Total	371	100.0
Household sizes	1–3 persons	143	38.5
	4–6 persons	173	46.6
	7–9 persons	39	10.5
	Over 9 persons	16	4.3
	Total	371	100.0

**Table 2:** Types of household pharmaceutical waste items.

Type of Pharmaceutical Wastes	Yes		No	
	F	%	F	%
Small plastic bags	252	67.9%	119	32.1%
Soft cardboard boxes	192	51.8%	179	48.2%
Small paper bags	158	42.6%	213	57.4%
Plastic bottles	182	49.1%	189	50.9%
Unused liquid-based medicines	57	15.4%	314	84.6%
Unused pills or tablets	109	29.4%	262	70.6%
Unused injections	13	3.5%	358	96.5%
Unused skin or wound-care creams	53	14.3%	318	85.7%
Unused portable health-checking devices	12	3.2%	359	96.8%

**Table 3:** Storage methods for pharmaceuticals.

Storage Place	Level of Agreement				
	Strongly Disagree	Disagree	Neither Disagree Nor Agree	Agree	Strongly Agree
I store them inside a medicine box	78 (21.1%)	41 (11.1%)	61 (16.4%)	81 (21.8%)	110 (29.6%)
I store them inside my handbag	125 (33.7%)	69 (18.6%)	64 (17.3%)	82 (22.1%)	31 (8.3%)
I store them everywhere in the house	207 (55.8%)	58 (15.6%)	44 (11.9%)	43 (11.6%)	19 (5.1%)
I store them in the bathroom cabinet	201 (54.2%)	40 (10.8%)	57 (15.4%)	46 (12.4%)	27 (7.2%)
I store them in the kitchen cabinet	123 (33.2%)	40 (10.8%)	55 (14.8%)	72 (19.4%)	81 (21.8%)
I store them in locked shelves	166 (44.7%)	59 (15.9%)	70 (18.9%)	36 (9.7%)	40 (10.8%)
I store them inside fridge	152 (41%)	39 (10.5%)	63 (17.0%)	67 (18.1%)	50 (13.4%)

**Table 4:** Disposal practices for pharmaceuticals.

Disposal Practice	Level of Agreement				
	Strongly Disagree	Disagree	Neither Disagree Nor Agree	Agree	Strongly Agree
I throw away it away into a domestic waste bin	68 (18.5%)	26 (7.5%)	42 (11.3%)	125 (33.4%)	110 (29.3%)
I throw it outside the house	204 (55.0%)	54 (14.6%)	38 (10.2%)	38 (10.2%)	37 (10.0%)
I flush it down the toilet drain	195 (52.5%)	43 (11.6%)	50 (13.5%)	41 (11.1%)	42 (11.3%)
I burn it outside where there is an open space	249 (67.1%)	51 (13.7%)	31 (8.4%)	22 (5.9%)	18 (4.9%)
I return it to hospitals and pharmacies	253 (68.2%)	48 (12.9%)	30 (8.1%)	18 (4.9%)	22 (5.9%)
I give it away to sick people	269 (72.5%)	41 (11.1%)	32 (8.6%)	16 (4.3%)	13 (3.5%)
I just keep it indefinitely in the household	157 (42.4%)	65 (17.5%)	65 (17.5%)	56 (15.1%)	28 (7.5%)

**Table 5:** Results on household willingness to return unwanted or unused medicines back to local pharmacies.

Household Willingness	Level of Agreement				
	Strongly Disagree	Disagree	Neither Disagree Nor Agree	Agree	Strongly Agree
I would return the unused medicine to the pharmacy	35 (9.4%)	24 (6.5%)	64 (17.3%)	97 (26.1%)	151 (40.7%)
I would highly recommend the programme to other people	29 (7.8%)	22 (5.9%)	69 (18.6%)	95 (25.6%)	156 (42.1%)
I intend to continue using the same disposal method rather than doing something different	143 (38.5%)	70 (18.9%)	79 (21.3%)	47 (12.7%)	32 (8.6%)
I intend to follow the advice given to me by health providers	21 (5.6%)	17 (4.6%)	70 (18.9%)	98 (26.4%)	165 (44.4%)

**Table 6:** Results from the ANOVA regarding household awareness about HPWs and demographical properties of households. Df = degree of freedom; F. = F value; sig. = significance (*p* value).

Demographic Variable		Sum of Squares	Df	Mean Square	F	Sig.
Gender	Between Groups	0.000	1	0.000	0.002	0.969
	Within Groups	65.525	369	0.178		
	Total	65.526	370			
Age	Between Groups	1.772	5	0.354	2.029	0.074
	Within Groups	63.756	365	0.175		
	Total	65.526	370			
Employment status	Between Groups	2.600	5	0.520	3.016	0.011
	Within Groups	62.926	365	0.172		
	Total	65.526	370			
Educational level	Between Groups	2.498	5	0.500	2.894	0.014
	Within Groups	63.027	365	0.173		
	Total	65.526	370			
Income level	Between Groups	0.969	5	0.192	1.084	0.369
	Within Groups	64.567	365	0.177		
	Total	65.526	370			
Place of residence	Between Groups	3.063	7	0.438	2.543	0.014
	Within Groups	62.463	363	0.172		
	Total	65.526	370			
Household type	Between Groups	1.084	6	0.181	1.020	0.412
	Within Groups	64.442	364	0.177		
	Total	65.526	370			
Household size	Between Groups	0.525	8	0.066	0.366	0.938
	Within Groups	65.001	362	0.180		
	Total	65.526	370			

**Table 7:** Results from ANOVA regarding classes of pharmaceutical wastes and households’ income, household size and places of residence. Df = degree of freedom; F = F value; Sig. = significance (*p* value).

Variable		Sum of Squares	Df	Mean Square	F	Sig.
Income level	Between Groups	31.430	5	6.286	2.365	0.039
	Within Groups	970.095	365	2.658		
	Total	1001.526	370			
Places of residence	Between Groups	16.443	7	2.349	0.866	0.534
	Within Groups	985.083	363	2.714		
	Total	1001.526	370			
Household size	Between Groups	38.062	8	4.758	1.788	0.078
	Within Groups	963.464	362	2.662		
	Total	1001.526	370			

**Table 8:** Results from ANOVA on household willingness to return pharmaceutical wastes according to age, education, gender and places of residence. Df = degree of freedom; F = F value; Sig. = significance (*p* value).

Variable		Sum of Squares	Df	Mean Square	F	Sig.
Gender	Between Groups	0.994	1	0.994	1.327	0.250
	Within Groups	276.348	369	0.749		
	Total	277.342	370			
Age	Between Groups	1.433	5	0.287	0.379	0.863
	Within Groups	275.909	365	0.756		
	Total	277.342	370			
Education	Between Groups	24.965	4	6.241	3.055	0.017
	Within Groups	747.769	366	2.043		
	Total	772.733	370			
Places of residence	Between Groups	4.342	7	0.620	0.825	0.567
	Within Groups	273.000	363	0.752		
	Total	277.342	370			

**Table 9:** The results of the ANOVA regarding perceptions of respondents in terms of waste disposal practices. Df = degree of freedom; F = F value; Sig. = significance (*p* value).

Variable		Sum of Squares	Df	Mean Square	F	Sig.
Throw away it away into household domestic waste bin	Between Groups	29.290	1	29.290	14.538	0.000
	Within Groups	743.443	369	2.015		
	Total	772.733	370			
Throw it outside the house	Between Groups	1.447	1	1.447	0.737	0.391
	Within Groups	724.365	369	1.963		
	Total	725.811	370			
I flush it down the toilet drain	Between Groups	9.833	1	9.833	4.734	0.030
	Within Groups	766.469	369	2.077		
	Total	776.302	370			
I burn it outside where there is an open	Between Groups	2.198	1	2.198	1.658	0.199
	Within Groups	488.988	369	1.325		
	Total	491.186	370			
I return it to hospitals and pharmacies	Between Groups	44.089	1	44.089	34.656	0.000
	Within Groups	469.447	369	1.272		
	Total	513.536	370			
I give it way to sick people	Between Groups	0.003	1	0.003	0.002	0.962
	Within Groups	407.723	369	1.105		
	Total	407.725	370			
I just keep it indefinitely	Between Groups	2.247	1	2.247	1.244	0.265
	Within Groups	666.600	369	1.807		
	Total	668.846	370			

## Data Availability

Data collected and analysed are available upon request.
